# Association between low-concentration PM_2.5_ exposure and emergency department visits for cardiovascular diseases: a time-series study

**DOI:** 10.3389/fpubh.2025.1704279

**Published:** 2025-12-10

**Authors:** Jiadong Zhang, Ying Wang, Zhao Li, Jichao Peng, Lifeng Dai, Nan Li, Yang Yi, Xiaoran Liu

**Affiliations:** 1 The First Affiliated Hospital of Hainan Medical University, Haikou, Hainan, China; 2 Key Laboratory of Hainan Trauma and Disaster Rescue, The First Affiliated Hospital of Hainan Medical University, Haikou, Hainan, China; 3 Key Laboratory of Emergency and Trauma Ministry of Education, Hainan Medical University, Haikou, Hainan, China; 4 Haikou Affiliated Hospital of Central South University Xiangya School of Medicine, Haikou, Hainan, China; 5 College of Public Health, Hainan Medical University, Haikou, Hainan, China; 6 College of Public Health, Southeast University, Nanjing, Jiangsu, China

**Keywords:** PM_2.5_, circulatory system diseases, air pollution, time series study, emergency department visits

## Abstract

**Introduction:**

Substantial epidemiological evidence suggests that both short- and long-term exposure to fine particulate matter (PM₂.₅) increases cardiovascular disease (CVD) risk. However, uncertainty persists regarding the cardiovascular effects of low-level PM₂.₅ exposure. This study aimed to clarify the association between PM₂.₅ and CVD morbidity in Haikou, China.

**Methods:**

A time-series design with Distributed Lag Non-linear Models (DLNMs) was employed to assess the short-term associations between PM₂.₅ exposure and daily CVD-related emergency department (ED) visits across three major hospitals in Haikou (2018–2021), with stratified analyses by sex and age.

**Results:**

Among 988,020 total ED visits, 69,099 (7.0%) were CVD-related. Our analyses revealed a distinctive three-phase, S-shaped nonlinear association between short-term PM₂.₅ exposure and emergency CVD visits, characterized by pronounced lag effects. Specifically, the risk of CVD exhibited an initial decline, followed by an increase and subsequent attenuation at higher concentrations.

**Discussion:**

We propose that this complex pattern reflects a balance between adaptive hormetic responses at low exposures, toxic effects at moderate levels, and behavioral adaptations during high pollution episodes. These findings underscore that maintaining PM₂.₅ concentrations within a moderate range may yield greater public health benefits in low-pollution regions like Haikou, offering critical guidance for refining air quality standards and preventive interventions.

## 1 Introduction

Atmospheric fine particulate matter (PM₂.₅)—airborne particles with an aerodynamic diameter ≤2.5 μm—has become a leading global environmental health risk factor (1, 2). Its minute size, extensive surface area, and strong adsorption capacity enable it to carry toxic substances, penetrate the respiratory barrier, and enter systemic circulation, thereby exerting profound physiological effects. Growing epidemiological evidence indicates that both short- and long-term PM₂.₅ exposure not only aggravates respiratory diseases but also substantially increases the incidence and mortality of cardiovascular and cerebrovascular diseases (CVD) (3, 4). The underlying mechanisms involve systemic inflammation, oxidative stress, endothelial dysfunction, hypercoagulability, and autonomic nervous system dysregulation (5–7). Although ambient air pollution remains a modifiable CVD risk factor, uncertainties persist regarding the cardiovascular health impacts of the relatively low-level PM₂.₅ exposure increasingly common in China and other regions. To address this gap, the present study conducted a detailed investigation in Haikou, a tropical island city in China.

## 2 Methods

### 2.1 Study area

Haikou, the capital of Hainan Province, functions as the province’s political, economic, technological, cultural, and transportation hub. Situated in northern Hainan Island (19°31′–20°04′N, 110°07′–110°42′E), the city lies within the tropical northern margin and experiences a marine monsoon climate characterized by warm, humid conditions, long summers, and the absence of a true winter. The climate features abundant rainfall, high solar radiation, minimal temperature variation, and an extended frost-free period. By the end of 2021, Haikou had a resident population of 2.908 million.

### 2.2 Data sources

**Figure 1:**
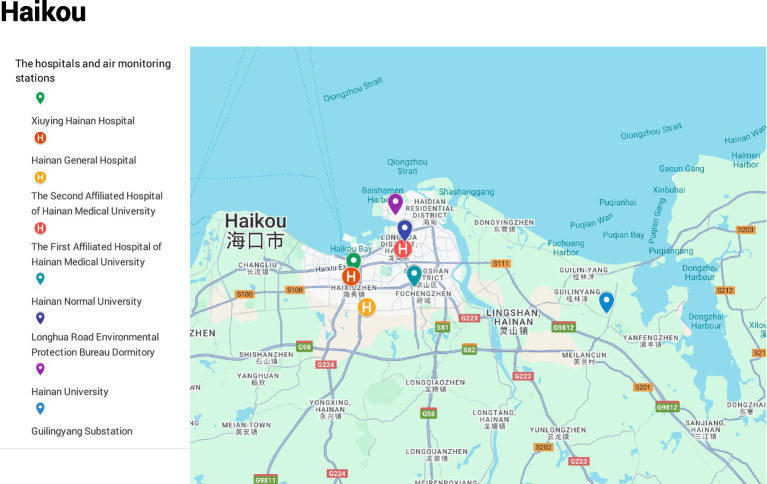
Study area map of Haikou city, showing the locations of the three hospitals and the five air quality monitoring stations. *Base map data: ©2025 Google*.

A map of the study area and data source locations is presented in Figure 1. Case data were obtained from the medical records departments of three Grade A tertiary hospitals: the First and Second Affiliated Hospitals of Hainan Medical University and Hainan General Hospital. Daily emergency department (ED) visit data from January 1, 2018, to December 31, 2021, were extracted, including patient demographics (name, age, sex), visit date, diagnosis, and residential address. Records lacking complete information were excluded to ensure data quality. In total, 988,020 ED visits were included. According to ICD-10 classifications (I00–I99), 69,099 visits were attributed to cardiovascular diseases, comprising 18,667 for hypertensive diseases (I10–I15), 9,799 for ischemic heart disease (I20–I25), and 32,212 for cerebrovascular disease (I60–I69).

Daily meteorological and air pollutant data for the study period (January 1, 2018–December 31, 2021) were collected. Meteorological variables—including daily mean temperature (°C), wind speed (m/s), sunshine duration (h), relative humidity (%), and atmospheric pressure (hPa)—were obtained from the Hainan Meteorological Bureau.

Air pollutant data were retrieved from the China Air Quality Online Monitoring and Analysis Platform^1^ and represented averaged measurements from five fixed monitoring stations: Longhua Road Environmental Protection Bureau Dormitory, Xiuying Hainan Hospital, Hainan University, Guilingyang Substation, and Hainan Normal University. The monitored pollutants included nitrogen dioxide (NO₂), ozone (O₃), sulfur dioxide (SO₂), carbon monoxide (CO), and particulate matter (PM₂.₅ and PM₁₀). Daily mean concentrations were used for NO₂, SO₂, CO, PM₂.₅, and PM₁₀, while O₃ levels were represented as the maximum 8-h moving average.

### 2.3 Statistical analysis

Statistical analyses were performed using multiple software platforms. Initial data cleaning and preprocessing were conducted in Microsoft Excel 2019. Descriptive statistics for emergency department (ED) visits, meteorological variables, and air pollutants—including temporal trends, demographic characteristics, and distributional features—were analyzed using SPSS 22.0. Normality testing indicated that daily CVD-related ED visits, pollutant concentrations, and meteorological variables followed non-normal distributions. Spearman correlation analyses were then performed in RStudio to identify significant correlations between ED visits and environmental factors for inclusion in multivariable models. Exposure–response relationships between environmental factors and daily CVD-related ED visits were estimated using distributed lag nonlinear models (DLNMs) with a quasi-Poisson distribution in R (version 4.2.0), employing the mgcv., splines, dlnm, and ggplot2 packages in accordance with established methods (8). Covariates included: (1) a natural cubic spline of calendar time (8 df/year) to adjust for long-term and seasonal trends; (2) indicator variables for day of the week; (3) meteorological factors; and (4) co-pollutants. To reduce multicollinearity, variables showing strong correlations with PM₂.₅ (|r| ≥ 0.7) were excluded from concurrent models. The DLNM structure was defined as Equation 1:


(1) $$\begin{array}{l} \log[E(Y_{t})]=\alpha +\beta X_{t,l}+\mathrm{ns}(\mathrm{Tim}e_{t},\mathrm{df}*\text{year})\\ +\sum \mathrm{ns}\;(X_{i},\mathrm{df})+\mathrm{as}.\text{factor}(\mathrm{do}w_{t}) \end{array}$$

where *Yₜ* denotes the observed daily ED visit count on day t, *E(Yₜ)* is the expected count, *α* is the intercept, *Xₜ,ₗ* is the cross-basis exposure matrix (with lag l), *β* is the vector of coefficients, ns() represents natural cubic splines (degrees of freedom specified per covariate), *Timeₜ* captures secular trends, *Xᵢ* denotes confounders, and *as.factor(dowₜ)* accounts for day-of-week effects. All continuous covariates—including temperature, relative humidity, sunshine hours, wind speed, air pressure, and concentrations of NO₂, SO₂, CO, and O₃—were modeled using natural cubic splines with 3 degrees of freedom to capture potential nonlinearities. Model fit was optimized by minimizing the quasi-Akaike Information Criterion (QAIC) and referencing prior cardiovascular epidemiological studies (9, 10). Sensitivity analyses were conducted by varying the degrees of freedom for the calendar time spline (7–10 per year). Relative risks (RRs) with 95% confidence intervals (CIs) were estimated relative to the median exposure level (P₅₀). All statistical tests were two-sided, and significance was defined as *p* < 0.05.

## 3 Results

### 3.1 Emergency department visits for cardiovascular diseases

From 2018 to 2021, a total of 69,099 CVD-related emergency department visits were recorded across the three hospitals in Haikou, with a median of 46 visits per day. Stratified median daily visits were as follows: ≥65 years (25 visits), 18–64 years (21 visits), 0–17 years (0 visits), males (28 visits), and females (18 visits). Patients aged ≥65 years accounted for 54.09% of all cases, and 60.04% were male. Among CVD subtypes, cerebrovascular disease represented the largest proportion (46.6%), followed by hypertensive (14.2%) and ischemic heart disease (14.2%). Detailed statistics are presented in Table 1.

**Table 1:** Emergency department visits for cardiovascular diseases in Haikou (2018–2021).

Variables	N	(%)	Min	*M* (*P*_25_, *P*_75_)	Max
ICD (I00-99)					
0–17 years old	1,110	1.61	0	0, 1	9
18–64 years old	30,611	44.30	3	16, 25	45
≥65 years old	37,378	54.09	4	18, 32	62
Male	41,485	60.04	5	22, 34	65
Female	27,614	39.96	3	14, 23	44
Total	69,099	100.00	13	37, 56	98
ICD (I10-15)					
0–17 years old	19	0.10	0	0, 0	2
18–64 years old	8,845	47.40	0	4, 8	23
≥65 years old	9,803	52.52	0	4, 9	30
Male	9,609	51.48	0	4, 9	22
Female	9,058	48.52	0	4, 8	26
Total	18,667	100.00	0	8, 16	48
ICD (I20-25)					
0–17 years old	9	0.01	0	0, 0	2
18–64 years old	4,190	42.76	0	1, 4	11
≥65 years old	5,600	57.15	0	2, 5	16
Male	6,647	67.83	0	3, 6	15
Female	3,152	32.17	0	1, 3	8
Total	9,799	100.00	0	4, 9	20
ICD (I60-69)					
0–17 years old	165	0.51	0	10, 17	2
18–64 years old	13,478	41.84	0	6, 12	32
≥65 years old	18,569	57.65	0	9, 16	34
Male	20,524	63.72	2	10, 17	36
Female	11,688	36.28	0	5, 10	26
Total	32,212	100.00	3	16, 27	58

N, number; P, percentile.

### 3.2 Air pollutants and meteorological data

Between 2018 and 2021, mean daily meteorological parameters in Haikou were: temperature 25.24 °C, precipitation 5.14 mm, relative humidity 80.33%, sunshine duration 5.31 h, wind speed 2.79 m/s, and atmospheric pressure 1004.00 hPa. Mean pollutant concentrations were: PM₂.₅ 15.67 μg/m^3^, PM₁₀ 30.16 μg/m^3^, NO₂ 11.38 μg/m^3^, SO₂ 4.62 μg/m^3^, CO 0.54 mg/m^3^, and O₃ 77.81 μg/m^3^. Daily PM₂.₅ levels exceeded China’s Grade II National Ambient Air Quality Standard (35 μg/m^3^) on only 5.5% of days (81 of 1,468). All annual mean pollutant concentrations met Grade I standards, except PM₂.₅, which slightly surpassed the threshold (Grade I limits: PM₂.₅ 15 μg/m^3^, PM₁₀ 40 μg/m^3^, NO₂ 40 μg/m^3^, SO₂ 20 μg/m^3^, CO 4 mg/m^3^, O₃-8 h 100 μg/m^3^). Comprehensive data are provided in Table 2.

**Table 2:** Characteristics of meteorological variables and ambient air pollutants in Haikou (2018–2021).

Variables	Mean	Sd	Min	P50	Max	IQR
Meteorological factors						
Temperature (°C)	25.24	4.38	8.5	26.2	33	6.2
Rainfall (mm)	5.14	17.33	0	0	266.6	1.4
Relative Humidity (%)	80.33	8.20	36	81	99	11
Sun time (h)	5.31	3.86	0	5.6	12.8	7.4
Wind speed (m/s)	2.79	0.81	0.7	2.7	6.8	1.1
Air pressure (hpa)	1004.00	6.20	984.9	1004.2	1,019	9.7
Air pollutants						
PM_2.5_ (μg/m^3^)	15.67	9.30	0	13	62	10
PM_10_ (μg/m^3^)	30.16	13.22	0	27	115	16
NO_2_ (μg/m^3^)	11.38	4.80	2	11	41	6
SO_2_ (μg/m^3^)	4.62	1.82	2	4	23	1
CO (mg/m^3^)	0.54	0.14	0.2	0.5	1.2	0.2
O_3_ (μg/m^3^)	77.81	32.46	20	71	207	40

SD, standard deviation; P, percentile; IQR, interquartile range.

### 3.3 Temporal trends

Time-series analyses (Supplementary Figures 1–6) revealed pronounced seasonal variations in temperature, sunshine duration, and atmospheric pressure. PM₂.₅, PM₁₀, SO₂, CO, and O₃ displayed synchronous cyclical fluctuations, with higher concentrations during autumn and winter. Concurrently, total CVD emergency visits and those for major subtypes exhibited progressively increasing fluctuations over time.

### 3.4 Correlation analysis of air pollutants, meteorological parameters, and cardiovascular disease emergency visits

Significant correlations were observed between CVD emergency visits and environmental factors from 2018 to 2021. Overall visit counts correlated negatively with mean temperature, relative humidity, and sunshine duration (*p* < 0.05) but positively with all six air pollutants (*p* < 0.05). Age-specific trends: Pediatric visits (0–17 years) correlated significantly only with temperature, whereas geriatric patients (≥65 years) showed the strongest correlations across nearly all variables except NO₂. Disease-specific trends: Hypertensive disease correlated negatively with temperature, humidity, and sunshine, but positively with wind speed, atmospheric pressure, and all pollutants (*p* < 0.05). Ischemic heart disease correlated negatively with temperature and sunshine duration and positively with atmospheric pressure, PM₂.₅, PM₁₀, CO, and O₃ (*p* < 0.05). No significant associations were found with humidity, wind speed, NO₂, or SO₂. Cerebrovascular disease correlated negatively with temperature and humidity, and positively with atmospheric pressure, PM₂.₅, PM₁₀, and O₃ (*p* < 0.05). No significant associations were noted with sunshine, wind speed, NO₂, SO₂, or CO. Meteorological-pollutant interactions showed negative correlations between temperature and all pollutants except NO₂ (positive, *p* < 0.05); relative humidity correlated negatively with all pollutants except CO (positive, *p* < 0.05); and sunshine duration correlated negatively with PM₂.₅, PM₁₀, SO₂, and CO but positively with NO₂ (*p* < 0.05). Significant inter-pollutant correlations were also observed (*p* < 0.05). Complete results are presented in Supplementary Table 1.

### 3.5 Exposure-response relationships

**Figure 2:**
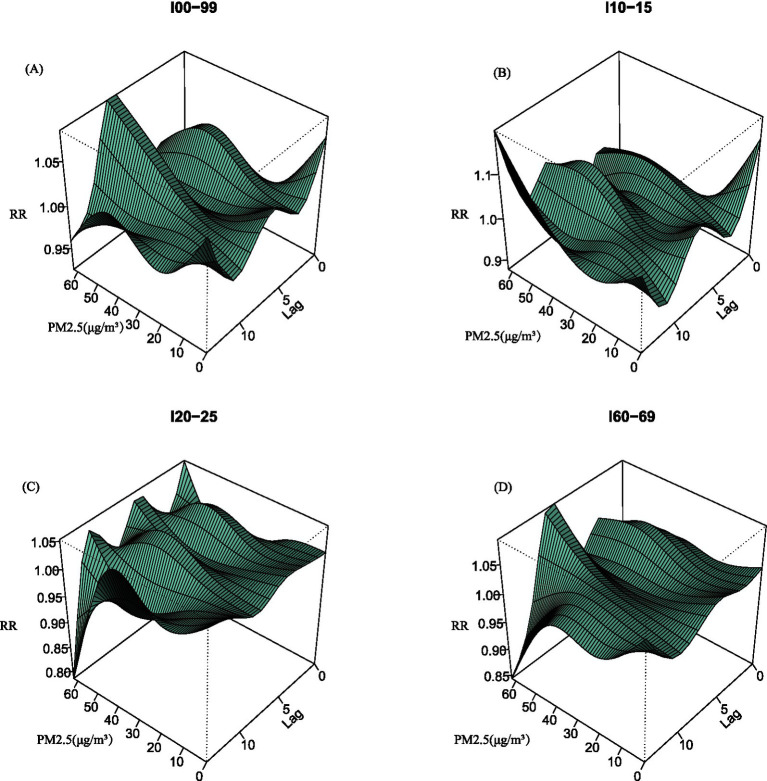
Three-dimensional effect surfaces of cardiovascular diseases across lag days using median exposure levels as reference.

Using distributed lag nonlinear models (DLNM), we assessed the spatiotemporal health impacts of PM₂.₅ exposure and visualized exposure–lag–response relationships through three-dimensional surface plots. The reference concentration was defined as 13 μg/m^3^ (the study median), with a maximum lag of 14 days to capture the full temporal effect. As shown in Figure 2, PM₂.₅ exhibited nonlinear associations with cardiovascular disease (CVD) emergency visits across the lag period. Peak risks appeared at distinct lag days for individual CVD subtypes when daily PM₂.₅ concentrations reached 62 μg/m^3^: overall CVD at lag 9 days (RR = 1.083, 95% CI: 0.987–1.188), hypertensive diseases at lag 14 days (RR = 1.193, 95% CI: 0.897–1.586), ischemic heart disease at lag 11 days (RR = 1.056, 95% CI: 0.839–1.329), and cerebrovascular disease at lag 8 days (RR = 1.092, 95% CI: 0.967–1.234).

**Figure 3:**
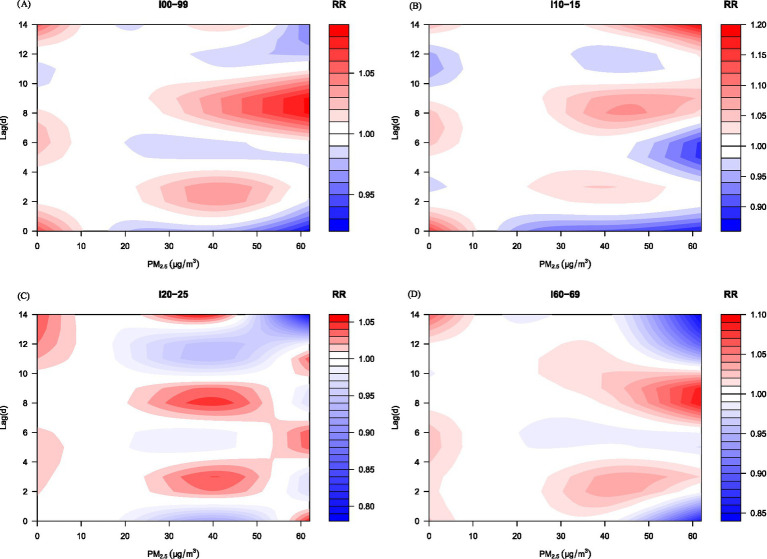
Contour maps of the impact of PM_2.5_ on emergency department visits for various circulatory system diseases under different lag days. The red area represents the risk effect, and the blue area represents the protective effect. The median of PM_2.5_ is considered as the reference value.

As illustrated in Figure 3, the effects of PM₂.₅ on CVD emergency visits manifested immediately at lag 0 and persisted throughout the 14-day period. During lags 0–1 days, elevated PM₂.₅ levels were associated with transient reductions in hospitalization risk. Between lags 2–4 days, risk began to rise progressively, reaching maximum intensity at lags 8–10 days, with a distinct peak at lag 9. Consistent temporal profiles were observed across CVD subtypes—hypertensive disorders, ischemic heart disease, and cerebrovascular events—mirroring the overall trend. Collectively, these findings highlight significant delayed and evolving effects of PM₂.₅ exposure on cardiovascular emergency visits, emphasizing the dynamic progression of risk over time.

**Figure 4:**
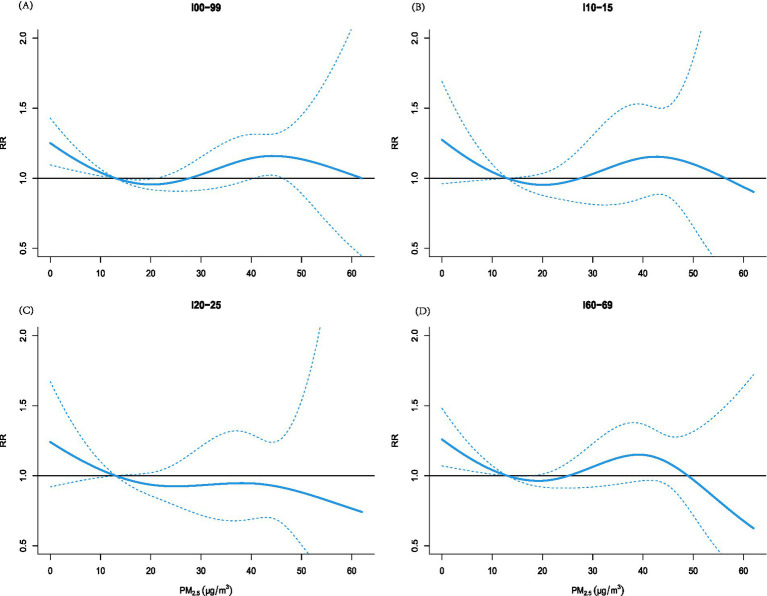
Exposure-effect curve (solid red line) and 95% confidence interval (gray area) of the relative risk of PM_2.5_ and visits to the emergency department for circulatory system diseases, with the median of PM_2.5_ as the reference value.

Figure 4 depicts S-shaped exposure–response curves between PM₂.₅ and CVD emergency visits across all subcategories. These were characterized by an initial decline in risk, a subsequent increase at moderate concentrations, and attenuation at higher exposure levels. Ischemic heart disease uniquely displayed a continuous risk reduction throughout the PM₂.₅ range. At 62 μg/m^3^, the lowest overall CVD risk was observed (RR = 0.854, 95% CI: 0.563–1.296), while a secondary risk peak occurred at 44 μg/m^3^ (RR = 1.159, 95% CI: 1.023–1.314). Lag-stratified analyses (Supplementary Table 2) revealed that at 5 μg/m^3^, overall CVD visits showed detrimental effects beginning at lag 0 and persisting cumulatively, whereas at 35 μg/m^3^, significant single-day effects emerged at lags 3 and 9 without cumulative significance. Hypertensive diseases showed isolated adverse effects at lag 9 (35 μg/m^3^) with no cumulative pattern, while ischemic heart disease remained unassociated across concentrations. Cerebrovascular disease displayed harmful effects at 5 μg/m^3^ that dissipated at 35 μg/m^3^, suggesting concentration-dependent effect modification among CVD subtypes.

**Figure 5:**
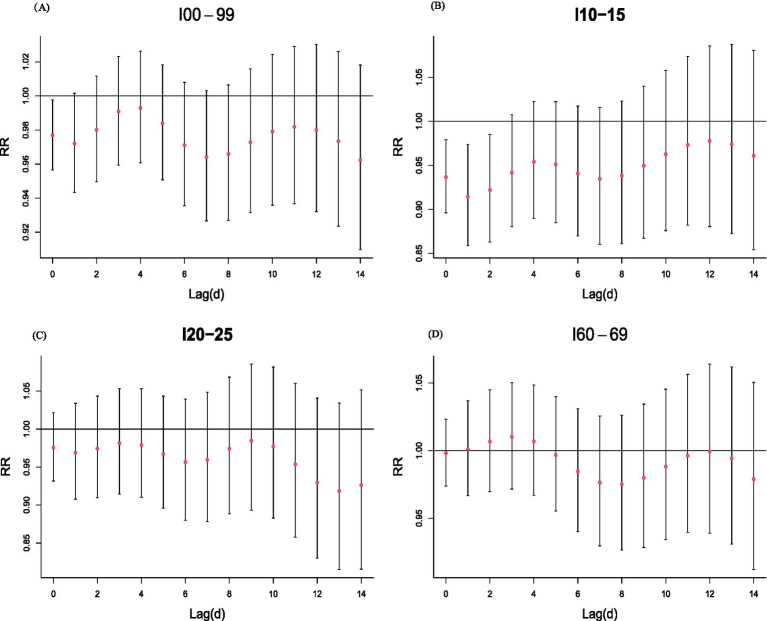
Association between PM_2.5_ and the risk of circulatory disease emergency visits (RR and 95% CI) across lag days, using the median PM_2.5_ as the reference value.

As shown in Figure 5, each 10 μg/m^3^ increase in daily PM₂.₅ during optimal lag periods corresponded to an 8.552% reduction in overall CVD emergency visits (95% CI: 2.631–14.113%), a 3.589% decrease in hypertensive disease visits (95% CI: −0.310 to 7.338%), and a 10.964% decline in ischemic heart disease visits (95% CI: −0.542 to 21.153%). In contrast, cerebrovascular disease exhibited a non-significant 1.165% increase (95% CI: −2.714 to 5.199%).

### 3.6 Sensitivity analysis

Sensitivity analyses (Supplementary Figures 7–10) demonstrated highly consistent 3D response surfaces across temporal covariate specifications (df = 7–10). The stability of these results confirms the robustness of the model and the reliability of the epidemiological associations.

## 4 Discussion

Our findings reveal a complex, three-stage S-shaped nonlinear relationship between short-term PM₂.₅ exposure at low concentrations and emergency department visits for circulatory system diseases in Haikou, accompanied by a significant lag effect. We propose that this distinctive pattern reflects the dynamic interplay of multiple biological and behavioral mechanisms across varying exposure intensities.

At very low PM₂.₅ concentrations, the initial decline in risk may reflect a hormetic response. In a relatively clean environment, a mild elevation in PM₂.₅ can act as a subtle stressor, activating protective biological processes. This includes transient increases in reactive oxygen species and inflammatory mediators that stimulate antioxidant defenses (e.g., superoxide dismutase, glutathione peroxidase) and anti-inflammatory signaling pathways (11, 12). Such adaptive mechanisms may transiently enhance cardiovascular resilience, manifesting as a slight, counterintuitive reduction in observed risk.

As concentrations rise to moderate levels, the toxic effects of PM₂.₅ exceed physiological compensatory capacity. Heightened systemic inflammation, oxidative stress, endothelial dysfunction, and autonomic imbalance sharply elevate the likelihood of acute cardiovascular events (12). Conversely, the subsequent plateau or decline in risk observed at higher concentrations may result from behavioral adaptations, such as reduced outdoor activity and the use of protective measures during severely polluted conditions.

Although numerous studies have established a positive correlation between PM₂.₅ exposure and circulatory disease risk (9, 13–15), evidence remains inconsistent at low concentrations. A multicity analysis across 184 Chinese cities reported increased cardiovascular hospitalizations linked to PM₂.₅, yet its high average exposure levels limit relevance to low-pollution regions such as Haikou (16). In contrast, a large U. S. study found only weak, nonsignificant associations between PM₂.₅ concentrations—even those below WHO guidelines—and cardiovascular emergency visits (17). This aligns with our observations, suggesting that low-level PM₂.₅ exposure may be more strongly associated with severe cardiovascular outcomes than with milder clinical presentations.

Previous research has also yielded inconsistent findings regarding lag effects of PM₂.₅ exposure (18, 19). In our analysis, the risks for overall circulatory diseases and hypertension paradoxically declined during the initial exposure days, followed by a marked increase after several days’ delay. This intricate lag pattern may reflect delayed symptom onset and reduced mobility during high-pollution periods, which transiently suppress immediate healthcare utilization.

Several days may elapse between exposure, symptom manifestation, and medical consultation. While acute cardiovascular effects are more likely after high PM₂.₅ episodes, lower exposure levels may instead precipitate delayed physiological responses, contributing to the observed temporal heterogeneity in risk.

A study conducted in São Paulo, Brazil, examined the effects of fine particulate matter (PM₂.₅) on hospitalizations for ischemic heart disease and observed earlier responses among women (two days post-exposure), although relative risk differences between sexes were not statistically significant (20). Consistent with this, our analysis (Supplementary Figure 11) revealed that for total cardiovascular disease (CVD), hypertension, and cerebrovascular disease, females exhibited inflection points at higher PM₂.₅ concentrations than males, indicating greater male susceptibility to PM₂.₅ exposure. This finding aligns with observations from Beijing (21) and may reflect longer outdoor exposure durations among men and greater occupational vulnerability to environmental hazards such as industrial dust, chemicals, and noise—factors that could amplify PM₂.₅-related health risks.

As shown in Supplementary Figure 11, no significant differences were observed in overall circulatory disease visits between middle-aged (18–64 years) and older (≥65 years) populations. However, for hypertension, older individuals displayed flatter concentration–response curves than their middle-aged counterparts, likely due to age-related declines in neural responsiveness and diminished sensitivity to blood pressure elevations (22, 23). Many older adults remain asymptomatic (e.g., without headache or dizziness) even at blood pressures ≥160 mmHg, reducing motivation to seek medical care. For ischemic heart disease, middle-aged adults exhibited U-shaped risk curves, while the older adult demonstrated inverted U-shaped patterns. This may result from greater outdoor exposure among middle-aged individuals, which heightens vulnerability to high PM₂.₅ levels, whereas older adults typically stay indoors during pollution episodes, lowering acute exposure. In cerebrovascular disease, middle-aged adults showed stable risk patterns, while the older adult displayed S-shaped curves, possibly reflecting compromised vascular integrity in advanced age.

Seasonal analysis indicated higher emergency visit frequencies in winter and lower frequencies in summer, likely reflecting Haikou’s low pollution levels and the protective influence of warm temperatures. Spearman correlation analysis revealed significant negative associations between temperature or humidity and circulatory diseases, hypertension, and cerebrovascular disease (except for a nonsignificant humidity–ischemic heart disease correlation), suggesting attenuated PM₂.₅ risks at low concentrations. Previous studies have shown that heatwaves elevate cardiovascular morbidity and mortality (24) through mechanisms such as hemoconcentration from dehydration (25) and heat-stress-induced arrhythmias or cardiac arrest (26). In Haikou’s tropical island climate, high humidity mitigates extreme heat stress despite promoting perspiration, while long-term acclimatization encourages hydration and electrolyte-balance behaviors that offset thermal strain. Cold exposure also influences circulatory disease risk by activating sympathetic nerves and the renin–angiotensin system (27, 28). A time-series analysis across 272 Chinese cities identified 22.8 °C as the minimum-mortality temperature, with cold effects being more prolonged than heat-related impacts(2–3 days) (29). Non-optimal temperatures accounted for 14.33% of non-accidental deaths: 10.49% attributable to moderate cold (−1.4 to 22.8 °C), 2.08% to moderate heat (22.8–29.0 °C), 1.14% to extreme cold (−6.4 to −1.4 °C), and 0.63% to extreme heat (29.0–31.6 °C). Similarly, a Vietnamese study (2008–2012) identified 26 °C as the optimal temperature minimizing older adult cardiovascular hospitalizations (30). During our observation period, Haikou maintained mild conditions (mean: 25.24 °C; range: 8.0–33.0 °C) except for cooler winters, accounting for the seasonal rise in circulatory disease visits.

Spearman analysis also demonstrated significant negative correlations between relative humidity and emergency visits for total cardiovascular disease, hypertension, and cerebrovascular disease, as well as between humidity and all measured air pollutants. However, the relationship between humidity and cardiovascular outcomes remains contentious. A U. S. study of 63 million adults found that higher summer mean specific humidity correlated with increased hospitalization risks for cardiovascular, coronary, and cerebrovascular diseases (31). Williams and Robin proposed an optimal temperature–humidity envelope—defined by core temperature and 100% relative humidity—beyond which mucosal function deteriorates (32). Research in three Chinese cities similarly found that both low (<45%) and high (80–90%) relative humidity levels may elevate cardiovascular mortality in Beijing. Haikou’s tropical monsoon–marine climate, characterized by a mean annual relative humidity of 80.33%, thus provides novel insights into cardiovascular risk mechanisms under persistently high-humidity conditions.

Although elevated humidity is known to enhance secondary PM formation and gaseous pollutant chemistry (33), the synergistic effects of humidity and air pollutants on cardiovascular health warrant further investigation.

This study offers several strengths. It provides the first estimates of PM₂.₅-associated emergency-visit risks for cardiovascular diseases in Haikou, addressing a critical knowledge gap for tropical island environments. The results establish a scientific foundation for air-quality management at low pollution levels and contribute essential regional evidence to the global discourse on air pollution and cardiovascular health. Moreover, the use of emergency-visit data—more sensitive to acute PM₂.₅ effects than hospitalization records—enhances detection of short-term exposure impacts and strengthens the study’s public-health relevance.

This study has several limitations. First, the use of fixed-site monitoring data as a proxy for individual exposure may lead to exposure misclassification. Second, case records were derived exclusively from three Grade A tertiary hospitals in Haikou, excluding data from secondary or community healthcare facilities, thereby limiting the generalizability of our findings. Third, confounding factors such as smoking, alcohol use, occupational exposure, and socioeconomic status could not be adjusted for due to limited data availability. Consequently, further research is warranted to clarify both the short- and long-term effects of low-concentration PM₂.₅ exposure on CVD incidence.

In summary, this study identified a non-linear, S-shaped association—rather than a monotonic positive relationship—between low-level PM₂.₅ exposure and emergency visits for CVD in Haikou. Distinct susceptibility patterns were observed across age and sex subgroups. These results suggest that maintaining PM₂.₅ levels within an optimal range may yield greater public health benefits than continuous reductions to ultra-low concentrations. Our findings provide valuable evidence for refining PM₂.₅ air quality standards and guiding local policymakers in developing targeted preventive measures.

## Data Availability

The raw data supporting the conclusions of this article will be made available by the authors, without undue reservation.

## Footnotes

1
https://www.aqistudy.cn/historydata/
