# Supplementary material for: Association between low-concentration PM2.5 exposure and emergency department visits for cardiovascular diseases: a time-series study
Source: Front Public Health. 2025 Dec 10;13:1704279. doi: 10.3389/fpubh.2025.1704279 (PMC12728070; doi:10.3389/fpubh.2025.1704279)
Supplement: Supplementary file 1 [file Data_Sheet_1.docx]

**Supplementary materials**

**
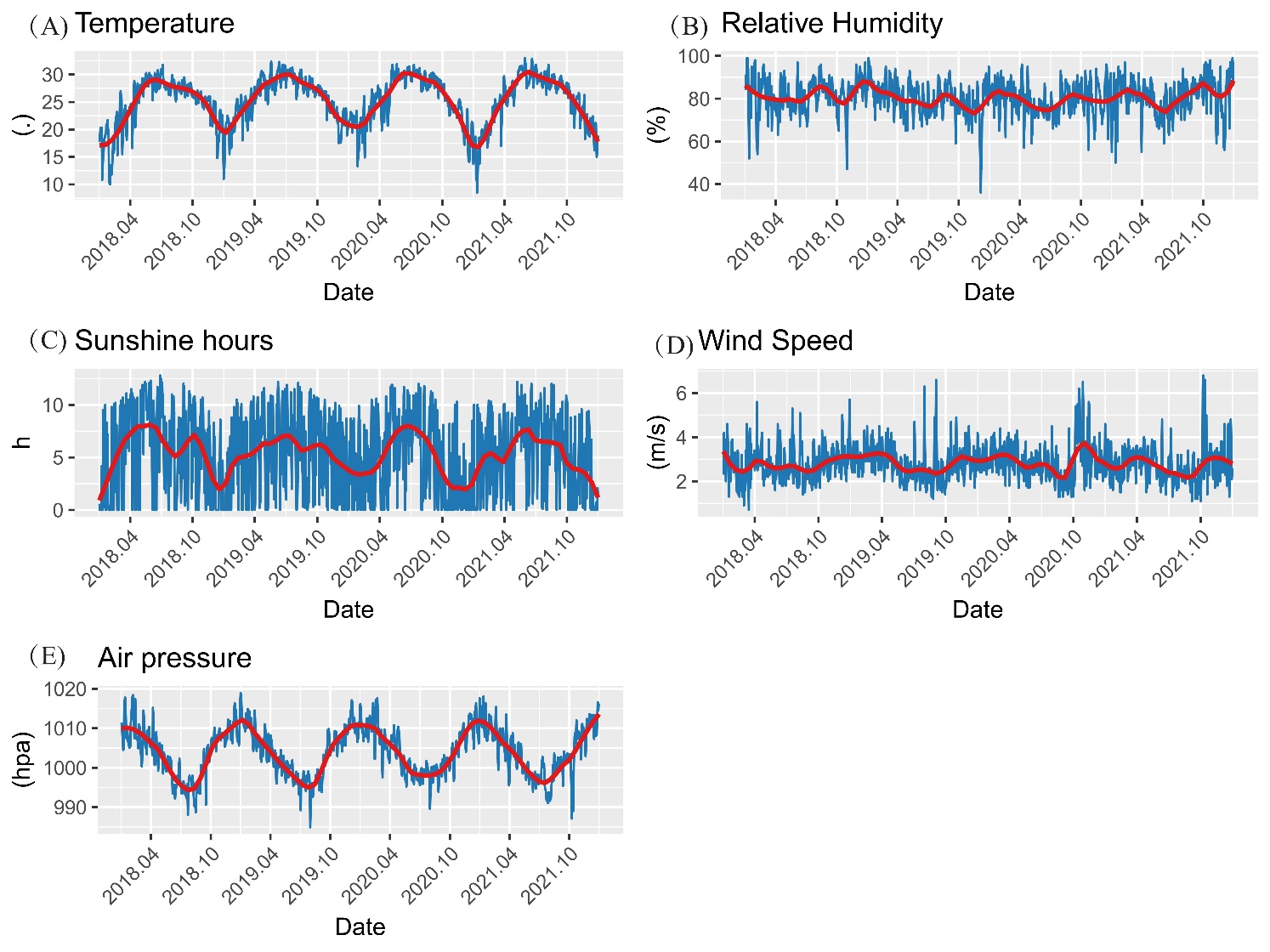
**

**Supplementary Figure 1.** Temporal patterns of the meteorological factors in Haikou, China, during 2018-2021.

**
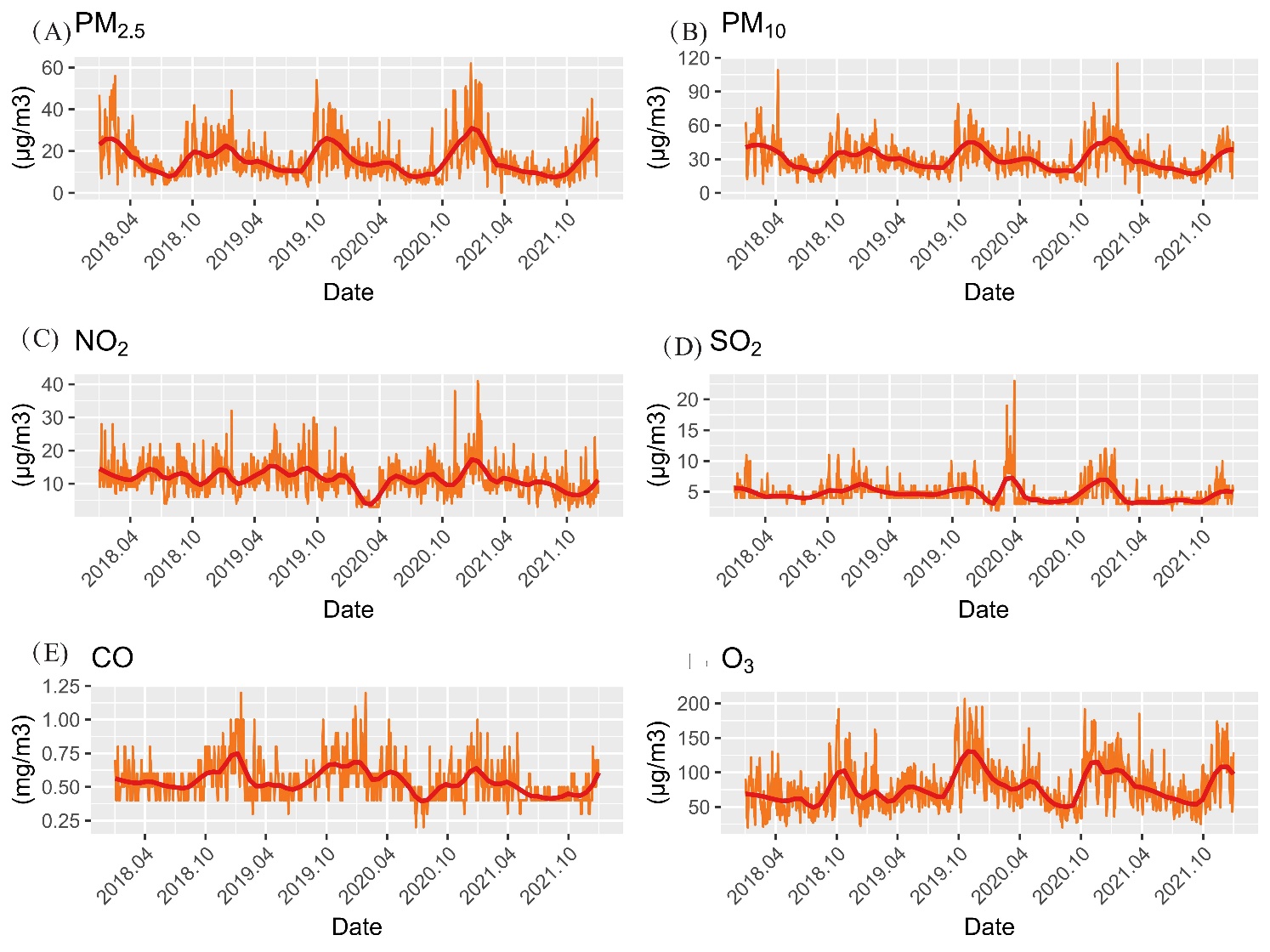
**

**Supplementary Figure 2.** Temporal patterns of the air pollutants in Haikou, China, during 2018-2021.

**
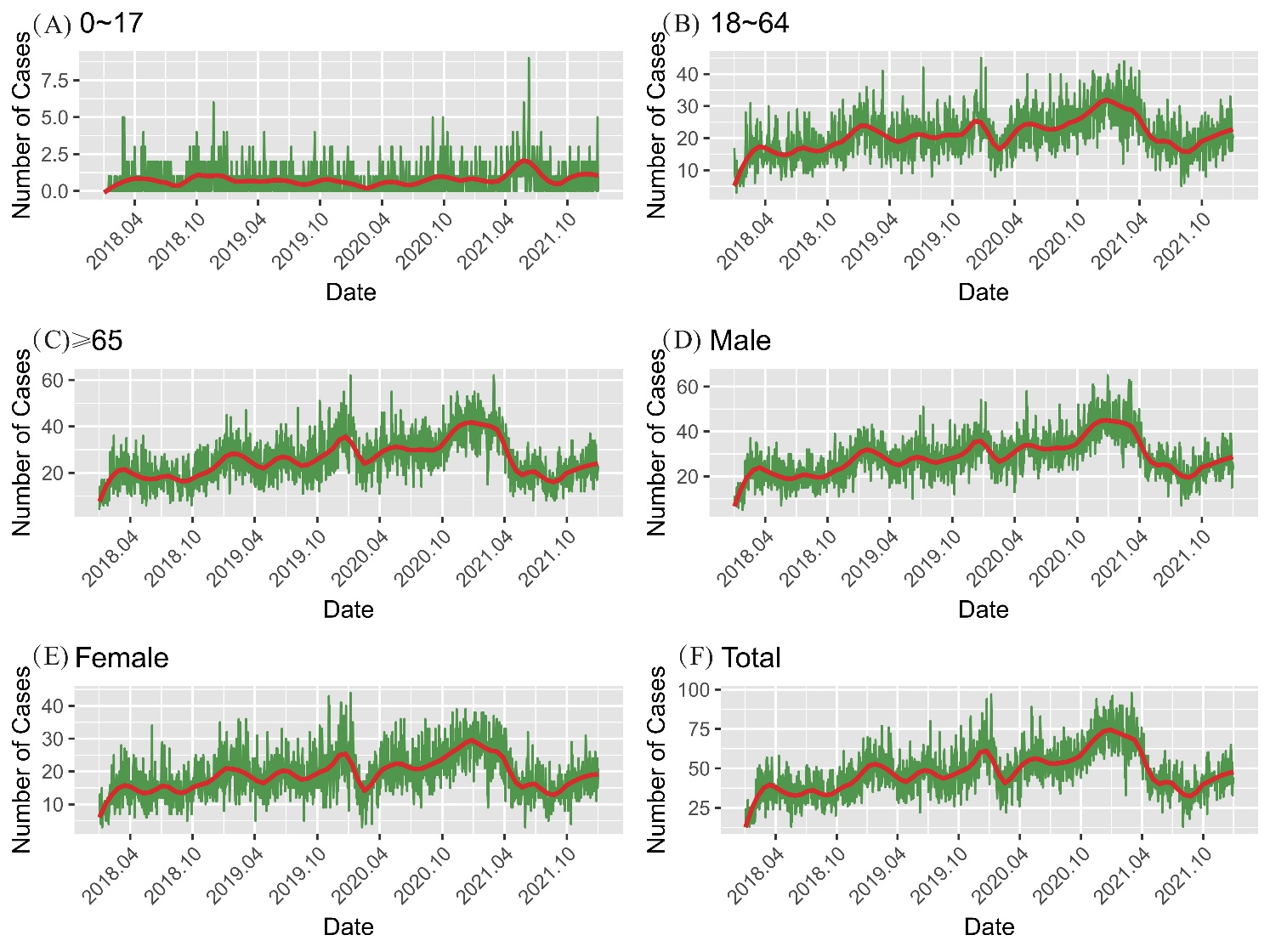
Supplementary Figure 3.** Temporal patterns of the emergency visits for ICD (0-99) in Haikou, China, during 2018-2021.

**
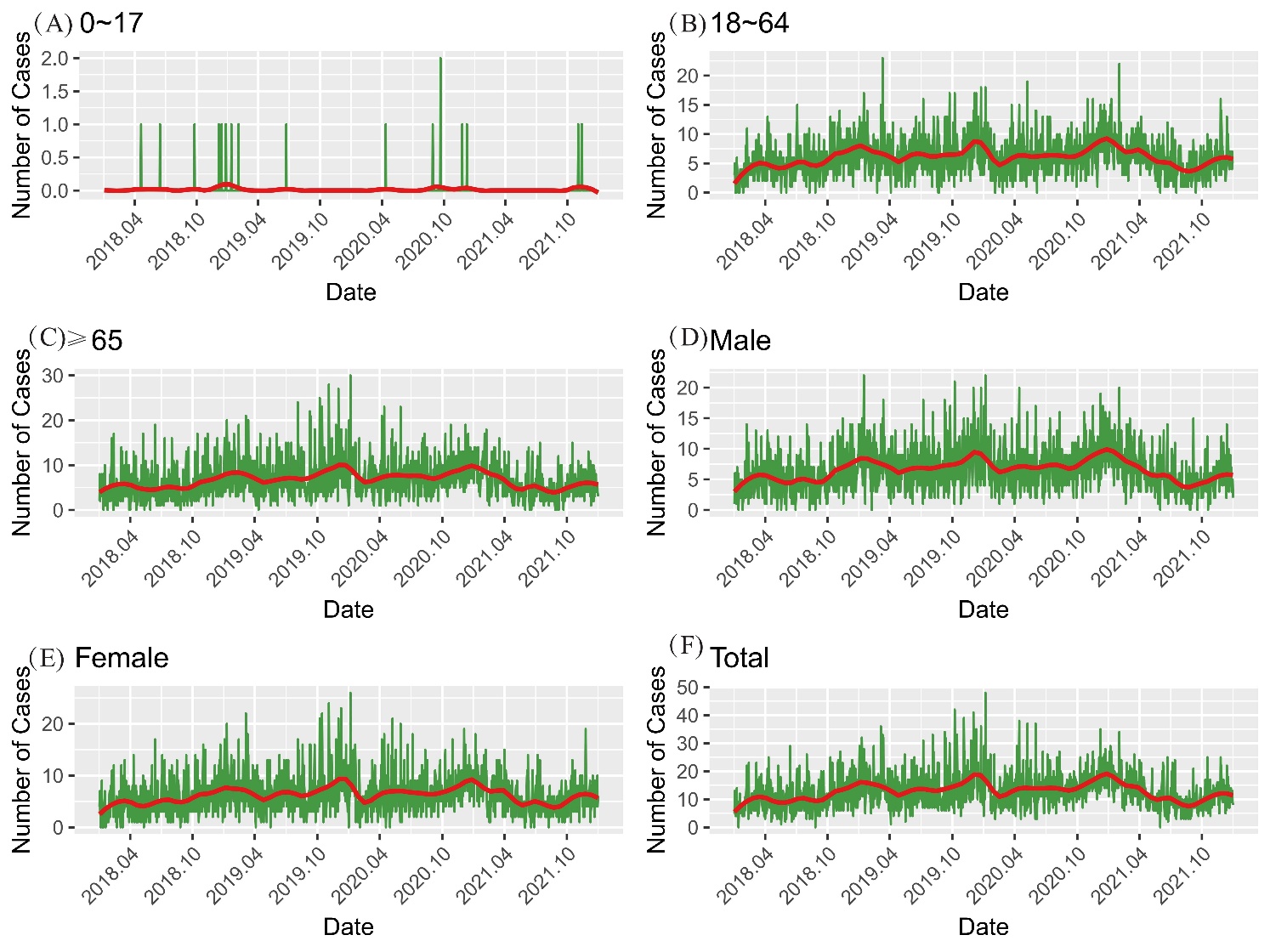
**

**Supplementary Figure 4.** Temporal patterns of the emergency visits for ICD (10-15) in Haikou, China, during 2018-2021.

**
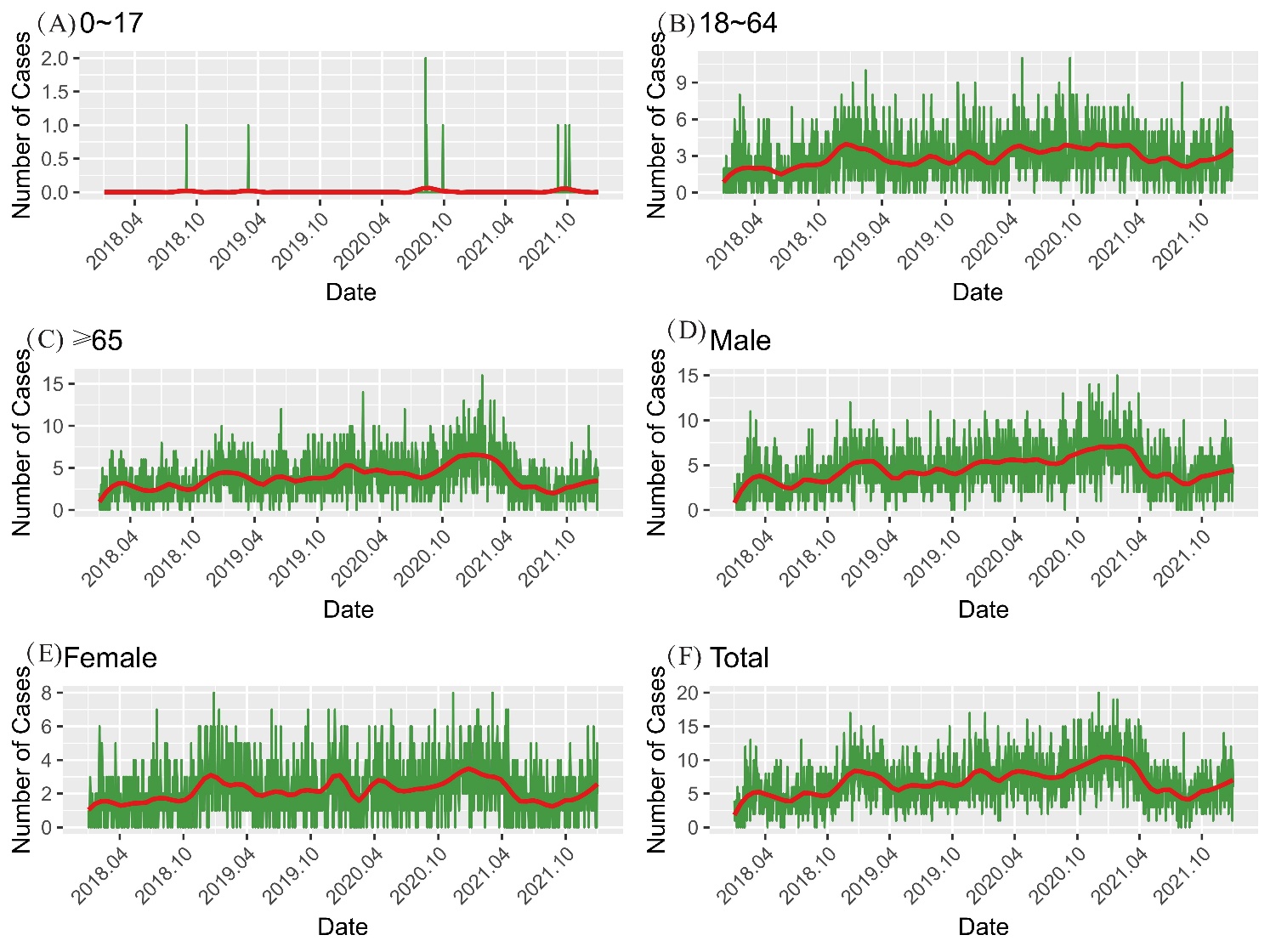
**

**Supplementary Figure 5.** Temporal patterns of the emergency visits for ICD (20-25) in Haikou, China, during 2018-2021.

**
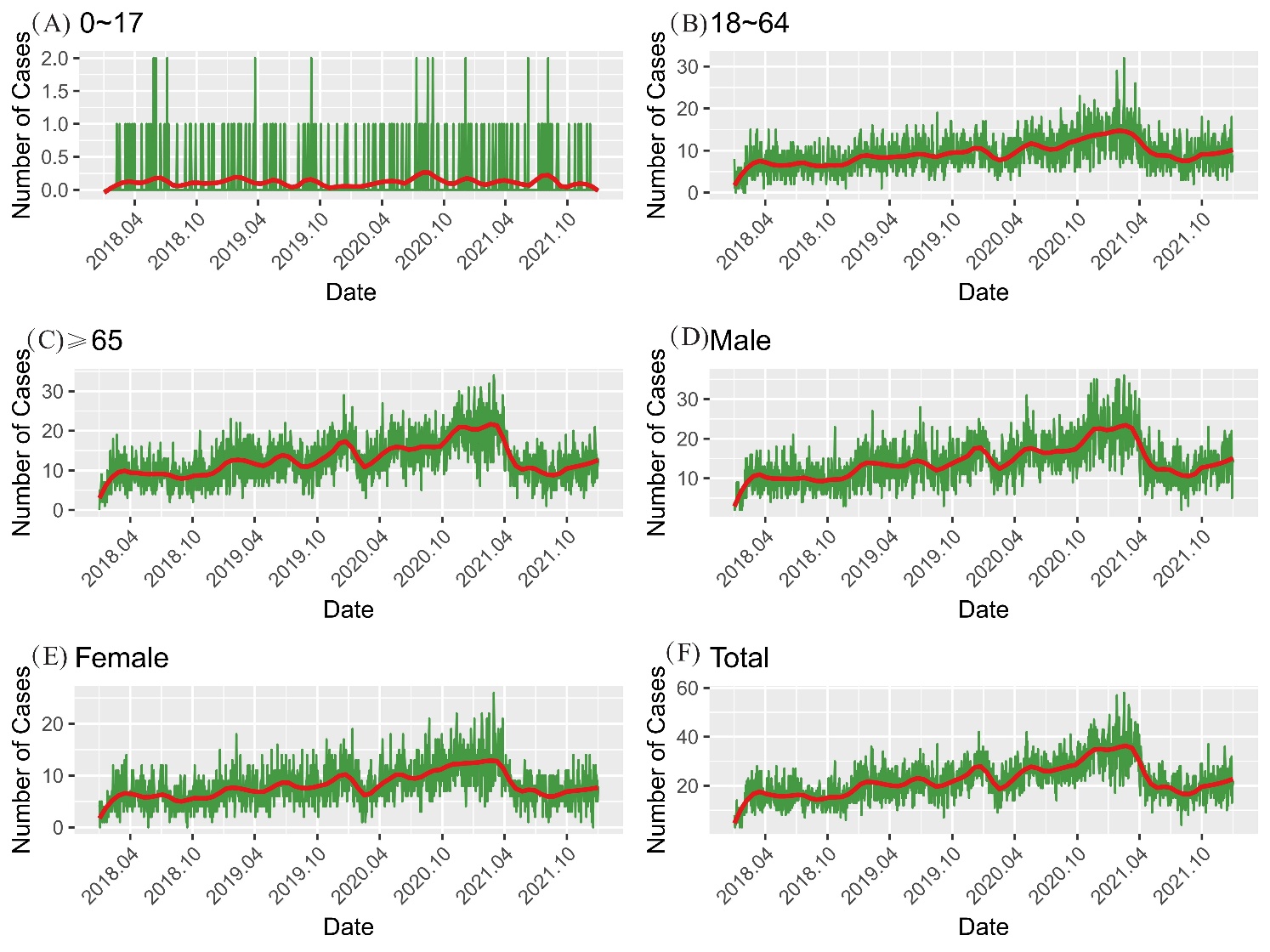
**

**Supplementary Figure 6.** Temporal patterns of the emergency visits for ICD (60-69) in Haikou, China, during 2018-2021.

**
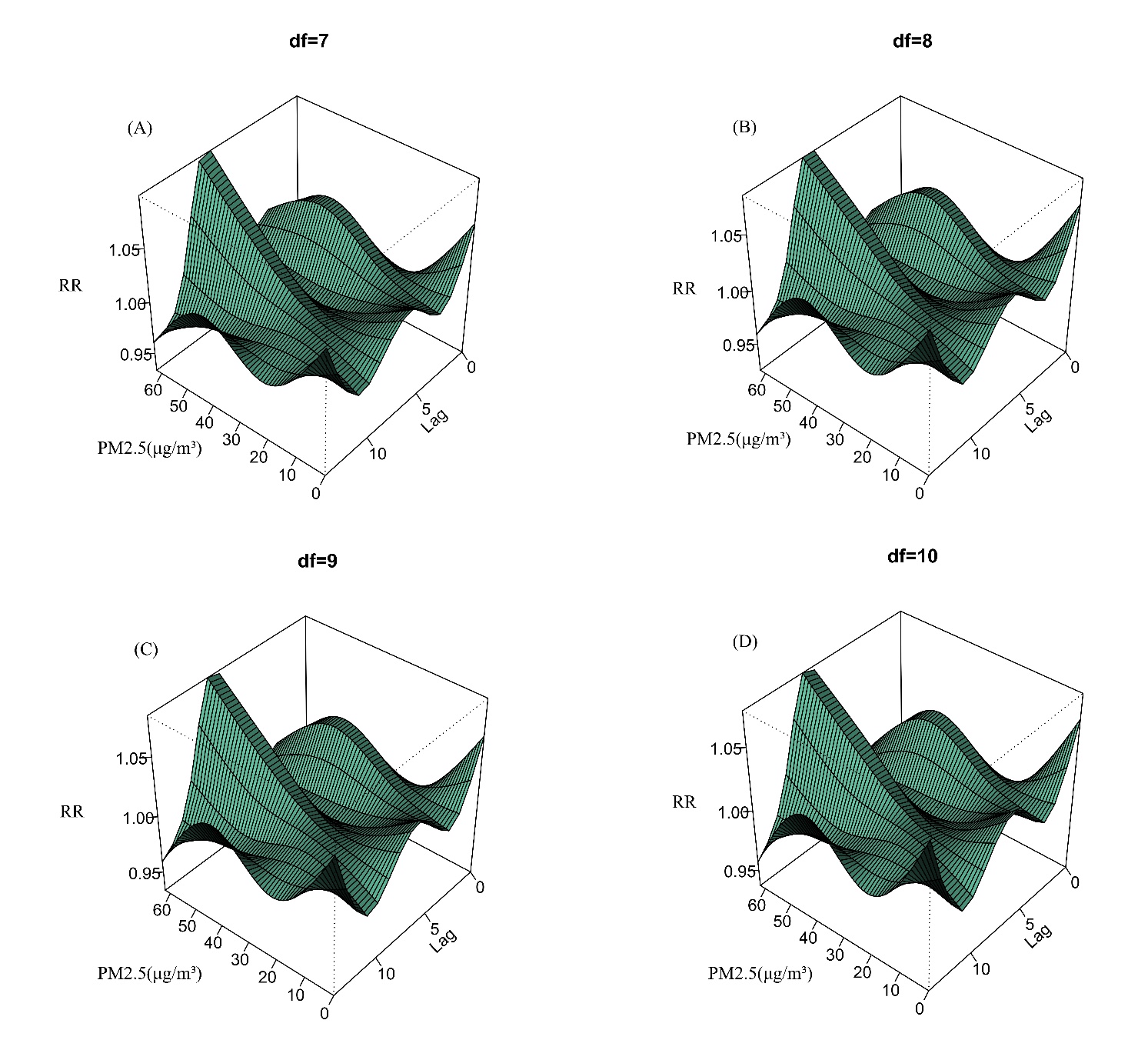
**

**Supplementary Figure 7.** Three-dimensional exposure-lag-response surface for PM_2.5_ and emergency department visits (ICD-10: I00-I99) using median exposure levels as the reference.

**
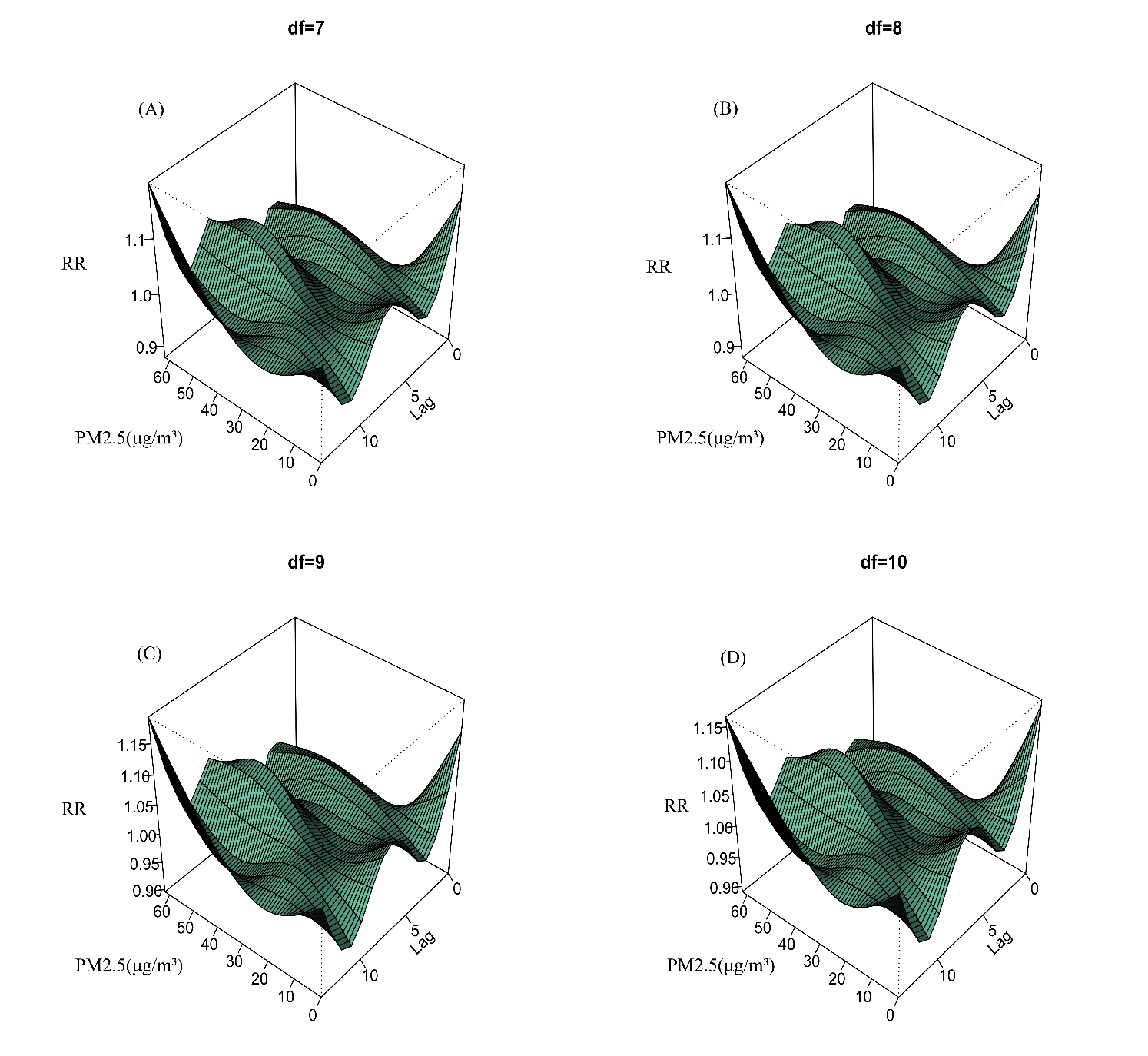
**

**Supplementary Figure 8.** Three-dimensional exposure-lag-response surface for PM_2.5_ and emergency department visits (ICD-10: I10-I15) using median exposure levels as the reference.

**
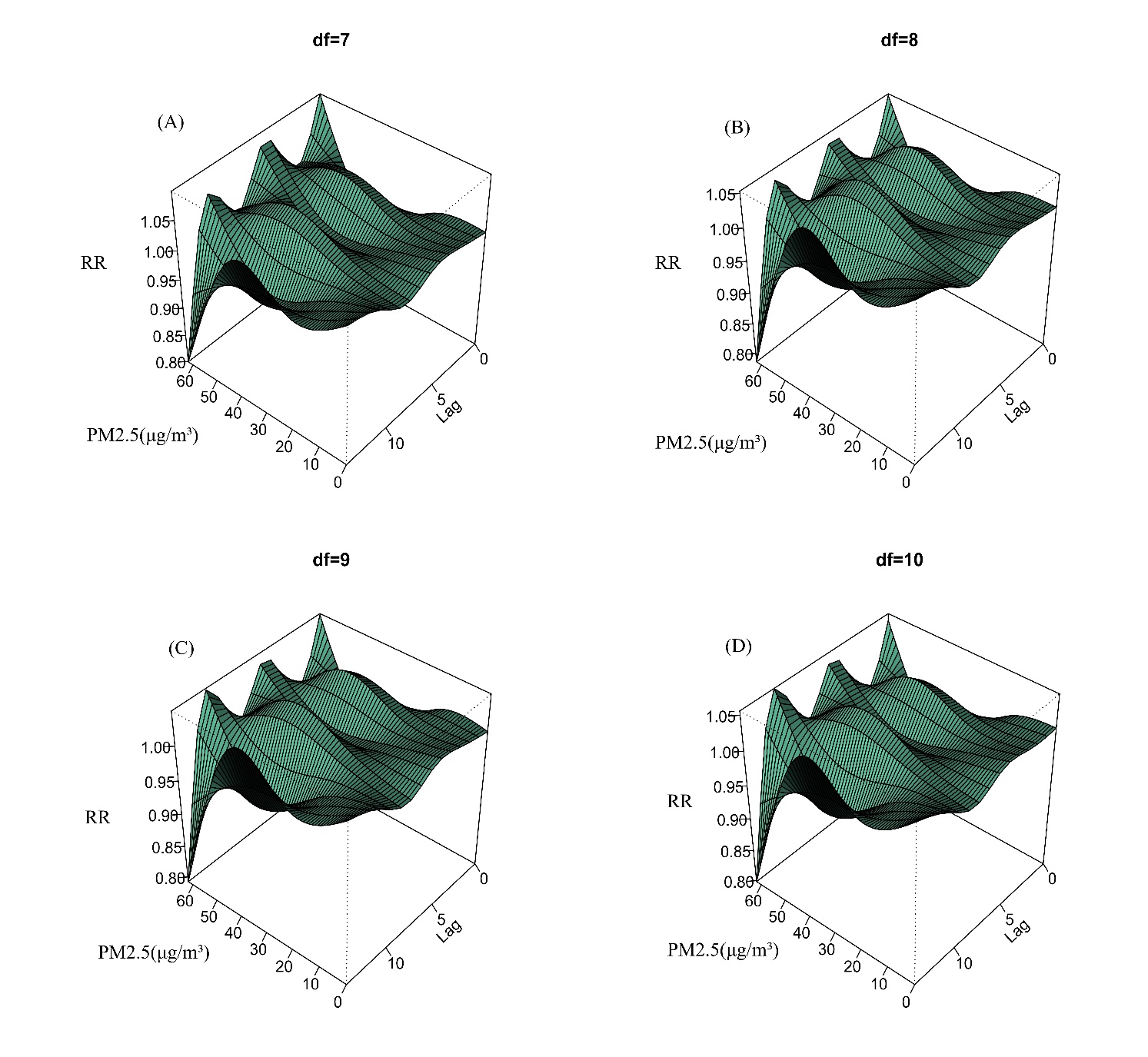
**

**Supplementary Figure 9.** Three-dimensional exposure-lag-response surface for PM_2.5_ and emergency department visits (ICD-10: I20-I25) using median exposure levels as the reference.

**
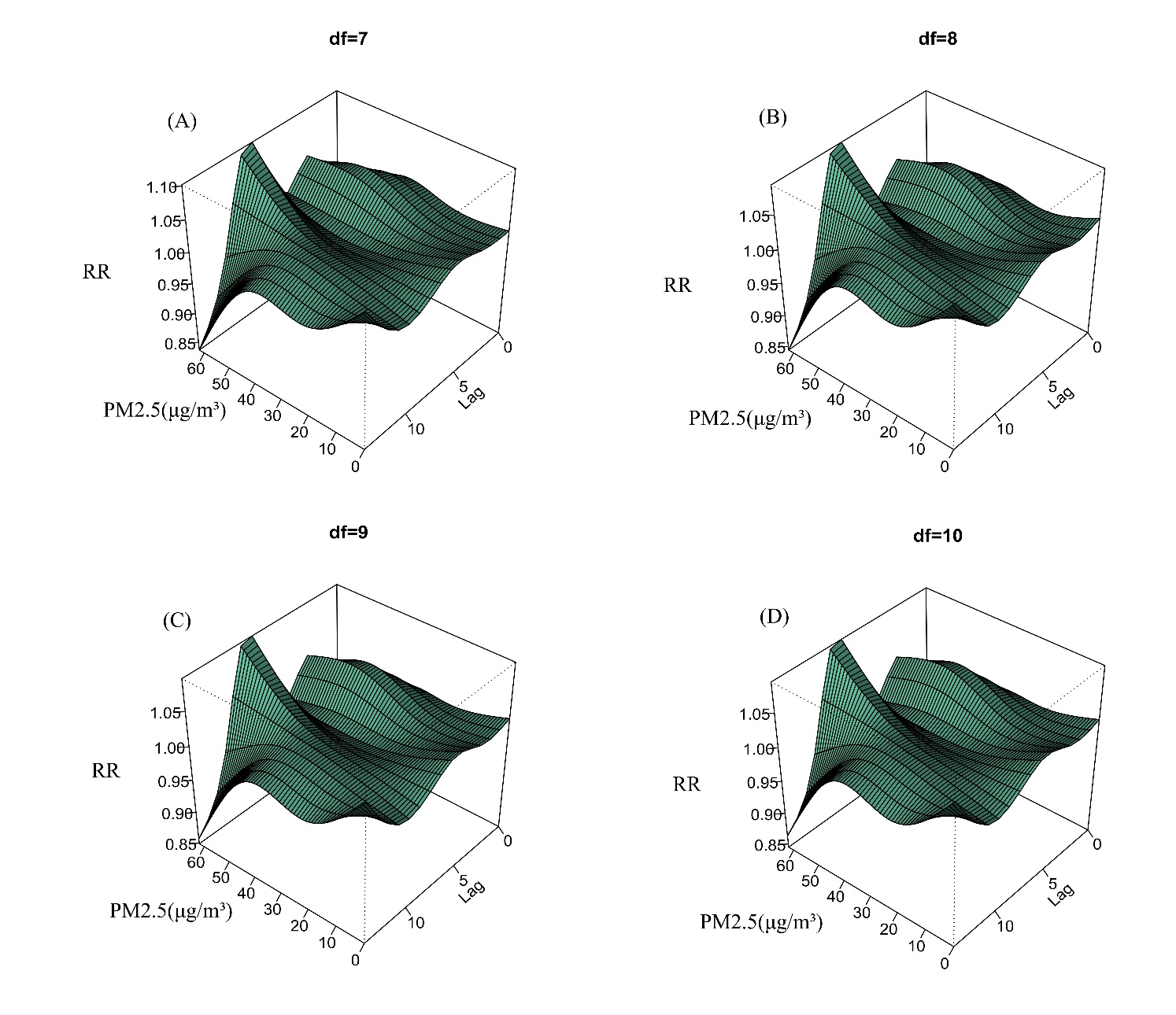
**

**Supplementary Figure 10.** Three-dimensional exposure-lag-response surface for PM_2.5_ and emergency department visits (ICD-10: I60-I69) using median exposure levels as the reference.

**
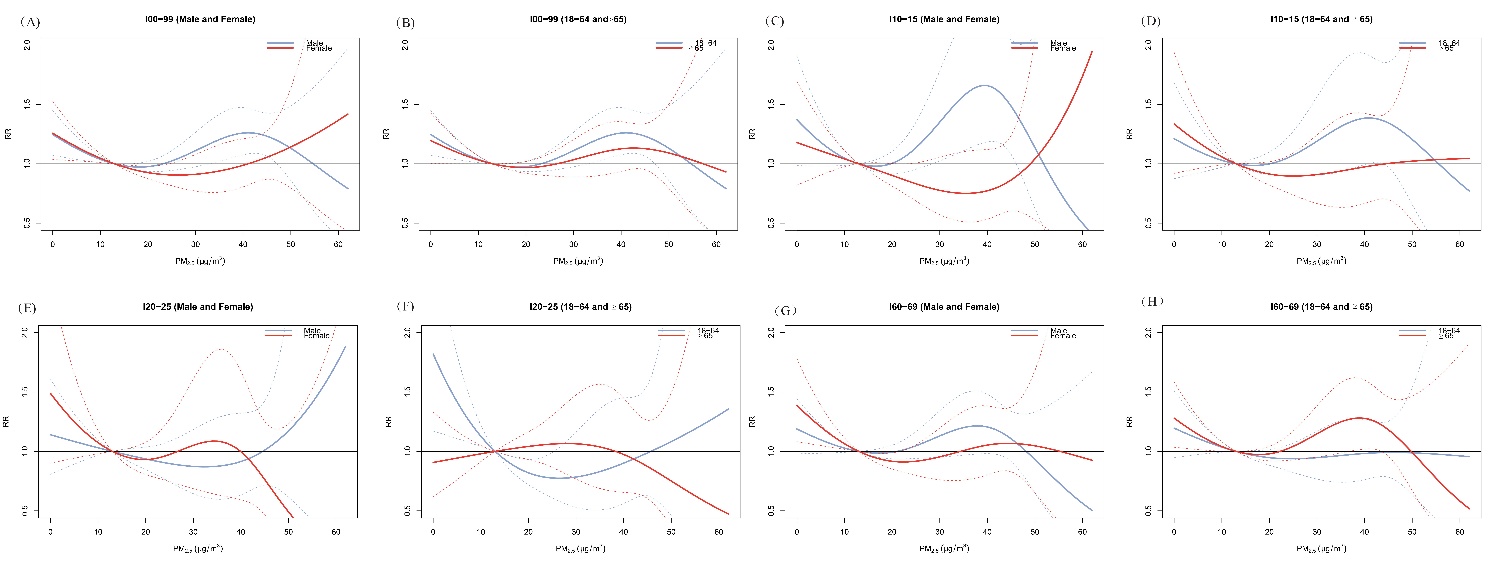
**

**Supplementary Figure 11.** Exposure-response curves of relative risks (RRs) and 95% confidence intervals for emergency department visits stratified by age and sex, using median exposure levels as the reference.

**Supplementary Table 1**

**Associations of air pollutants and meteorology with CVD emergency visits, Haikou 2018–2021**

|  | **Temperature** | **Relative humidity** | **Sun time** | **Wind speed** | **Air pressure** | **PM_2.5_** | **PM_10_** | **NO_2_** | **SO_2_** | **CO** | **O_3_** |
| --- | --- | --- | --- | --- | --- | --- | --- | --- | --- | --- | --- |
| ICD (I0-99) |  |  |  |  |  |  |  |  |  |  |  |
| Total | -0.179** | -0.063* | -0.091** | 0.057* | 0.251** | 0.162** | 0.199** | 0.080** | 0.063* | 0.129** | 0.260** |
| 0-17 years old | 0.069** | -0.021 | 0.029 | -0.05 | -0.004 | 0.015 | 0.017 | 0.029 | 0.005 | -0.006 | 0.002 |
| 18-64 years old | -0.128** | -0.042 | -0.063* | 0.024 | 0.191** | 0.132** | 0.158** | 0.086** | 0.056* | 0.109** | 0.215** |
| ≥65 years old | -0.192** | -0.064* | -0.096** | 0.075** | 0.251** | 0.157** | 0.197** | 0.061* | 0.064* | 0.123** | 0.250** |
| Male | -.181** | -0.060* | -0.086** | 0.057* | 0.256** | 0.158** | 0.188** | 0.059* | 0.048 | 0.109** | 0.238** |
| Female | -0.141** | -0.036 | -0.086** | 0.047 | 0.193** | 0.131** | 0.168** | 0.087** | 0.058* | 0.128** | 0.225** |
| ICD (I10-15) |  |  |  |  |  |  |  |  |  |  |  |
| Total | -0.193** | -0.066** | -0.095** | 0.076** | 0.232** | 0.151** | 0.191** | 0.099** | 0.136** | 0.172** | 0.215** |
| 0-17 years old | -0.032 | 0.017 | -0.019 | 0.004 | 0.040 | 0.027 | 0.034 | -0.015 | 0.044 | 0.028 | 0.022 |
| 18-64 years old | -0.144** | -0.063* | -0.057* | 0.031 | 0.191** | 0.161** | 0.188** | 0.116** | 0.132** | 0.181** | 0.208** |
| ≥65 years old | -0.180** | -0.047 | -0.104** | 0.086** | 0.193** | 0.096** | 0.134** | 0.059* | 0.095** | 0.118** | 0.151** |
| Male | -0.169** | -0.069** | -0.073** | 0.078** | 0.206** | 0.136** | 0.170** | 0.087** | 0.111** | 0.145** | 0.171** |
| Female | -0.158** | -0.035 | -0.091** | 0.043 | 0.185** | 0.116** | 0.151** | 0.079** | 0.114** | 0.148** | 0.185** |
| ICD (I20-25) |  |  |  |  |  |  |  |  |  |  |  |
| Total | -0.173** | 0.037 | -0.110** | 0.025 | 0.190** | 0.093** | 0.108** | 0.018 | 0.010 | 0.105** | 0.124** |
| 0-17 years old | 0.043 | 0.025 | 0.003 | -0.036 | -0.056* | -0.063* | -0.047 | -0.021 | -0.058* | -0.016 | -0.067* |
| 18-64 years old | -0.059* | 0.014 | -0.034 | -0.002 | 0.081** | 0.031 | 0.042 | 0.026 | -0.018 | 0.048 | 0.048 |
| ≥65 years old | -0.189** | 0.024 | -0.114** | 0.038 | 0.204** | 0.115** | 0.126** | 0.007 | 0.032 | 0.105** | 0.148** |
| Male | -0.148** | 0.028 | -0.083** | 0.002 | 0.162** | 0.078** | 0.084** | 0.002 | -0.016 | 0.078** | 0.104* |
| Female | -0.146** | 0.038 | -0.108** | 0.053* | 0.162** | 0.087** | 0.108** | 0.030 | 0.058* | 0.103** | 0.108** |
| ICD (I60-69) |  |  |  |  |  |  |  |  |  |  |  |
| Total | -0.075** | -0.099** | -0.34 | 0.021 | 0.149** | 0.092** | 0.123** | 0.037 | -0.017 | 0.042 | 0.229** |
| 0-17 years old | 0.036 | 0.000 | 0.011 | -0.019 | -0.014 | -0.041 | -0.043 | -0.008 | -0.010 | -0.035 | -0.027 |
| 18-64 years old | -0.037 | -0.061* | -0.029 | -0.005 | 0.087** | 0.038 | 0.056* | 0.019 | -0.047 | 0.009 | 0.158** |
| ≥65 years old | -0.092** | -0.101** | -0.033 | 0.032 | 0.159** | 0.115** | 0.148** | 0.043 | 0.014 | 0.055* | 0.231** |
| Male | -0.094** | -0.094** | -0.037 | 0.033 | 0.172** | 0.110** | 0.140** | 0.023 | -0.001 | 0.037 | 0.227** |
| Female | -0.032 | -0.066* | -0.016 | 0.002 | 0.074** | 0.042 | 0.064* | 0.034 | -0.048 | 0.026 | 0.156** |
| Temperature | 1 | -0.342** | 0.532** | -0.228** | -0.838** | -0.526** | -.0479** | 0.052* | -0.325** | -0.415** | -0.306** |
| Relative Humidity |  | 1 | -0.609** | 0.013 | 0.116** | -0.154** | -0.215** | -0.058* | -0.218** | 0.161** | -0.290** |
| Sun time |  |  | 1 | -0.282** | -0.326** | -0.089** | -0.057* | 0.107** | -0.061* | -0.344** | 0 |
| Wind speed |  |  |  | 1 | 0.246** | 0.019 | 0.087** | -0.473** | 0.014 | 0.039 | 0.016 |
| Air pressure |  |  |  |  | 1 | 0.594** | 0.576** | -0.113** | 0.330** | 0.368** | 0.417** |
| PM_2.5_ |  |  |  |  |  | 1 | 0.931** | 0.227** | 0.558** | 0.476** | 0.651** |
| PM_10_ |  |  |  |  |  |  | 1 | 0.199** | 0.542** | 0.420** | 0.676** |
| NO_2_ |  |  |  |  |  |  |  | 1 | 0.250** | 0.276** | 0.055* |
| SO_2_ |  |  |  |  |  |  |  |  | 1 | 0.395** | 0.393** |
| CO |  |  |  |  |  |  |  |  |  | 1 | 0.377** |
| O_3_ |  |  |  |  |  |  |  |  |  |  | 1 |

*P < 0.05, ** P < 0.01.

**Supplementary Table 2**

**Single-day and cumulative lag effects of daily PM_2.5_ exposure on cardiovascular disease emergency visits: relative risks (RR) and 95% confidence intervals**

|  | **I00-99** | | **I10-15** | | **I20-25** | | **I60-69** | |
| --- | --- | --- | --- | --- | --- | --- | --- | --- |
| Lag（d） | PM_2.5_(μg/m^3^) | | PM_2.5_(μg/m^3^) | | PM_2.5_(μg/m^3^) | | PM_2.5_(μg/m^3^) | |
|  | 5 | 35 | 5 | 35 | 5 | 35 | 5 | 35 |
| Single-lag |  |  |  |  |  |  |  |  |
| Lag 0 | 1.035（1.006，1.064）* | 0.977（0.932，1.024） | 1.084(1.022,1.149）* | 0.917(0.832,1.011） | 1.005（0.944，1.070） | 0.932（0.843，1.031） | 1.012（0.978，1.047） | 1.005（0.952，1.062） |
| Lag 1 | 1.016（1.003，1.030）* | 1.009（0.985，1.033） | 1.035(1.005,1.065）* | 0.976(0.930,1.025） | 1.004（0.975，1.036） | 0.986（0.937，1.039） | 1.008（0.992，1.025） | 1.020（0.992，1.049） |
| Lag 3 | 0.998（0.982，1.013） | 1.032（1.007，1.058）* | 0.982(0.950,1.015） | 1.040(0.988,1.094） | 1.007（0.972，1.044） | 1.035（0.980，1.093） | 1.008（0.988，1.027） | 1.025（0.995，1.057） |
| Lag 5 | 1.011（0.996，1.026） | 0.987（0.966，1.010） | 1.009(0.979,1.041） | 1.000(0.955,1.046） | 1.012（0.979，1.045） | 0.981（0.932，1.031） | 1.015（0.997，1.034） | 0.990（0.964，1.019） |
| Lag 7 | 1.013（1.002，1.026）* | 1.002（0.983，1.021） | 1.033(1.007,1.059）* | 1.032(0.993,1.072） | 1.001（0.975，1.028） | 1.013（0.972，1.056） | 1.009（0.995，1.024） | 0.991（0.968，1.015） |
| Lag 9 | 0.999（0.985，1.014） | 1.026（1.004，1.050）* | 1.006(0.976,1.038） | 1.053(1.006,1.103）* | 0.997（0.965，1.030） | 1.029（0.979，1.082） | 0.996（0.978，1.021） | 1.014（0.986，1.043） |
| Lag 11 | 0.991（0.976，1.006） | 0.994（0.971，1.018） | 0.968(0.936,1.000） | 0.981(0.934,1.030） | 1.013（0.978，1.049） | 1.013（0.896，1.049） | 0.995（0.976，1.014） | 1.016（0.987，1.046） |
| Lag 14 | 1.032（1.007，1.056）* | 1.013（0.976，1.051） | 1.033(0.982,1.086） | 1.019(0.945,1.100） | 1.021（0.967，1.079） | 1.021（0.968，1.079） | 1.035（1.005，1.066）* | 0.997（0.952，1.045） |
| Cumulative-lag |  |  |  |  |  |  |  |  |
| Lag 0~1 | 1.052（1.011，1.094）* | 0.986（0.922，1.055） | 1.121(1.032，1.218）* | 0.895(0.778，1.030） | 1.010（0.925，1.102） | 0.919（0.796，1.062） | 1.020（0.973，1.069） | 1.026（0.947，1.110） |
| Lag 0~3 | 1.052（1.007，1.100）* | 1.049（0.973，1.132） | 1.099(1.000，1.207） | 0.952(0.814，1.114） | 1.023（0.927，1.128） | 0.976（0.827，1.151） | 1.035（0.981，1.091） | 1.083（0.987，1.188） |
| Lag 0~5 | 1.066（1.017，1.118）* | 1.048（0.963，1.140） | 1.097(0.992，1.215） | 0.974(0.818，1.159） | 1.045（0.940，1.162） | 0.966（0.801，1.165） | 1.063（1.003，1.125）* | 1.082（0.974，1.202） |
| Lag 0~7 | 1.098（1.040，1.160）* | 1.033（0.936，1.139） | 1.164(1.036，1.308）* | 1.006(0.822，1.231） | 1.056（0.933，1.194） | 0.960（0.770，1.197） | 1.089（1.018，1.164）* | 1.055（0.933，1.194） |
| Lag 0~9 | 1.106（1.041，1.174）* | 1.084（0.973，1.208） | 1.202(1.056，1.367）* | 1.123(0.899，1.403） | 1.048（0.916，1.200） | 1.030（0.807，1.314） | 1.087（1.010，1.170）* | 1.075（0.938，1.231） |
| Lag 0~11 | 1.087（1.019，1.160）* | 1.090（0.969，1.227） | 1.142(0.995，1.311） | 1.120(0.878，1.429） | 1.067（0.923，1.232） | 0.957（0.734，1.249） | 1.074（0.993，1.162）* | 1.111（0.959，1.288） |
| Lag 0~14 | 1.134（1.053，1.222）* | 1.090（0.944，1.260） | 1.146(0.978，1.344） | 1.097(0.814，1.478） | 1.133（0.958，1.340） | 0.944（0.681，1.309） | 1.137（1.038，1.245）* | 1.127（0.940，1.352） |

The concentrations of 5 μg/m³ and 35 μg/m³ represent the 5th and 95th percentiles of daily PM_2.5_ levels in Haikou, China, respectively (*P < 0.05).
